# Range-wide phylogeography of the flightless steppe beetle *Lethrus apterus* (Geotrupidae) reveals recent arrival to the Pontic Steppes from the west

**DOI:** 10.1038/s41598-022-09007-0

**Published:** 2022-03-24

**Authors:** Gábor Sramkó, András Kosztolányi, Levente Laczkó, Rita Rácz, Lajos Szatmári, Zoltán Varga, Zoltán Barta

**Affiliations:** 1 MTA-DE ‘Lendület’ Evolutionary Phylogenomics Research Group, Debrecen, 4032 Hungary; 2 Department of Botany, University of Debrecen, Debrecen, 4032 Hungary; 3 Department of Ecology, University of Veterinary Medicine Budapest, Budapest, 1077 Hungary; 4 Department of Evolutionary Zoology and Human Biology, University of Debrecen, Debrecen, 4032 Hungary; 5 MTA-DE Behavioural Ecology Research Group, Department of Evolutionary Zoology and Human Biology, University of Debrecen, Debrecen, 4032 Hungary

**Keywords:** Population genetics, Phylogenetics, Entomology

## Abstract

The Eurasian Steppe belt is one of the largest biomes in the Northern Hemisphere. We provide here a range-wide phylogeography of the flightless steppe beetle *Lethrus apterus* that inhabits the western part of the Steppe belt through the study of population-level variance of mitochondrial cytochrome oxidase I sequences and nuclear microsatellites. We detected a concordant geographic structure of genetic data with a significant isolation-by-distance pattern. We found more genetic variation in the western part of the area and identified Northern Bulgaria and the Pannonian Basin as possible refugia. Genetic clusters were separated by main rivers in the eastern part of the area. This implies west-to-east colonisation and argues for an evolutionarily recent arrival of this species to its current main distribution area, the Pontic Steppes. This contradicts the classical biogeographical wisdom that assumed an east-to-west colonisation pattern.

## Introduction

The Eurasian steppe zone is one of the largest biomes on our planet covering approximately 13 million km^2^ from Eastern Central Europe in Hungary on the west to Manchuria in China on the east^1,2^. The western Eurasiatic part of this vast grassland zone is called the Euro-Siberian Steppe Zone (ESSZ)^3^ that reaches its westernmost edge in Europe represented by the Pannonian Steppes (i.e., the forest-steppe zone and extrazonal and edaphic steppes of the Pannonian Basin)^4^. The core area of the ESSZ in Eastern Europe is the Pontic Steppes (i.e., the steppe zone north of the Black Sea)^4^. The several dozens of animal and plant species showing a distribution pattern with presence in both the Pannonian and the Pontic Steppes are commonly called Pontic-Pannonian species and they represent a distinct distribution type in European biota^5,6^.

Under natural conditions, this biome is characterised by the overwhelming prevalence of grasslands with Poaceae species playing a major role in vegetation architecture^3^. As these grasslands produced one of the most fertile soils of our planet, the whole steppe region, by providing arable fields for grain production, has shrunken to habitat islands especially in the European part^1,2^. The remaining steppe patches, however, still host an outstanding biological diversity^7–9^. The vulnerability of the remaining steppe habitats, however, urgently calls for a better understanding of the structure, functional components and evolutionary history of this biome. Results obtained by phylogeographic studies can effectively contribute to the efficient management of threatened species^10^.

Phylogeography, the spatio-temporal genetic variation of populations of a species on its distribution area^11,12^, can inform us about important past processes that led to the formation of the species’ current distribution area. Past major geoclimatic events, for instance, had a strong influence on the phylogeographic structure of many species^13,14^. This especially holds for temperate European species strongly affected by the Pleistocene glaciations^15,16^ leading to genetic disparities within the species’ area. Describing the spatio-temporal genetic variations of such species, apart from its nature conservational value, is important for the general understanding of the biogeographic history of the temperate biota. While we have a thorough understanding of the phylogeography of several European temperate arboreal species including both plants and animals, similar information on steppe organisms is scarce^3^. Nevertheless, several steppe organisms reach the western edge of their distribution in Europe^17^, and this peripheral part of the area can also be important for the evolutionary history of even more easterly distributed species^18,19^.

**Figure 1:**
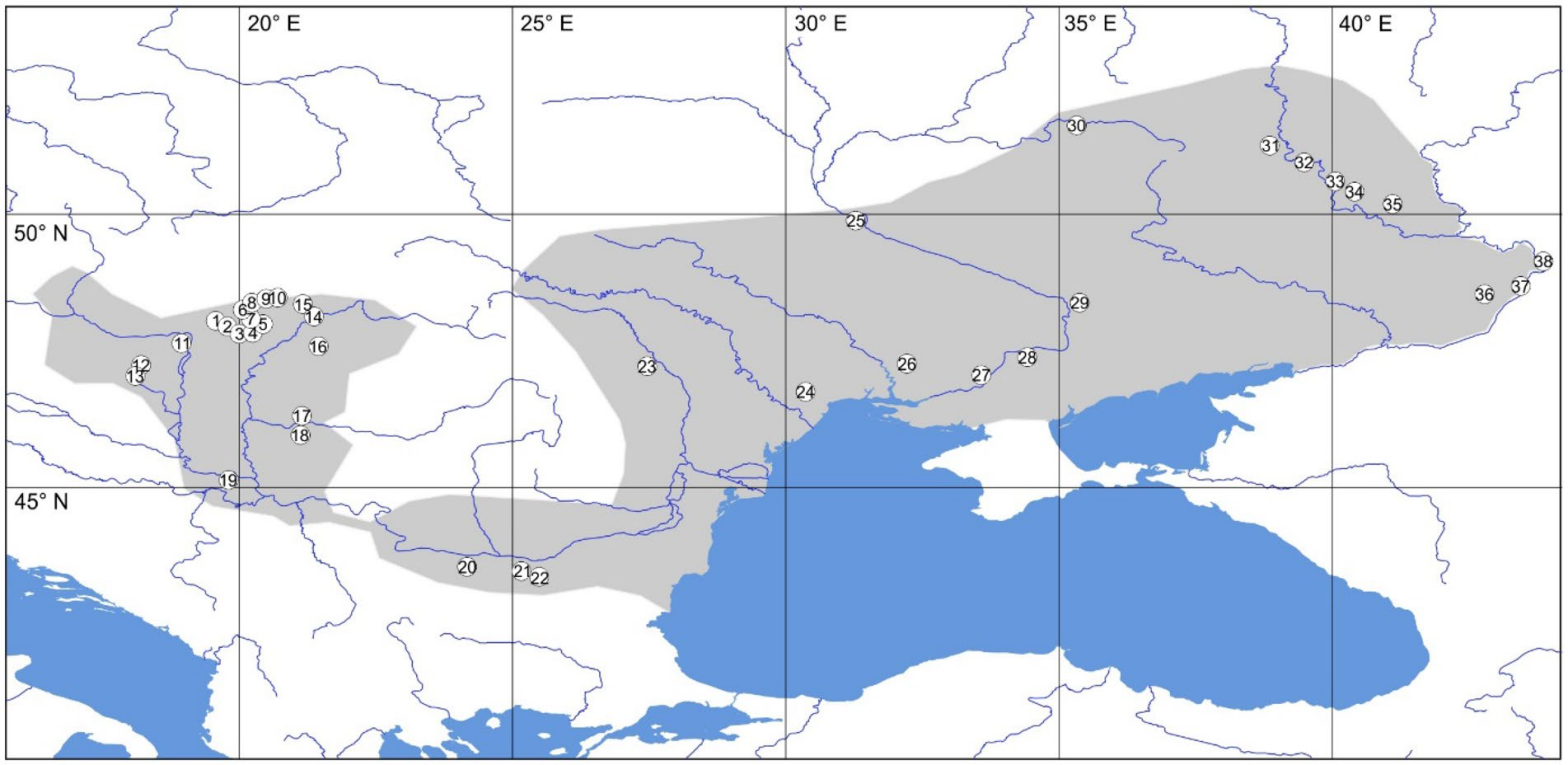
Distribution map of *Lethrus apterus* (shaded area) after Bagaturov and Nikolajev^24^, populations sampled for the current study are numbered according to Table 1. The map was created in QGIS v.3.10 ( http://www.qgis.org/ ) using geospatial layers downloaded from the freely available dataset Natural Earth ( https://www.naturalearthdata.com/ ).

Species with limited dispersal abilities are good candidates for a phylogeographic analyses as their molecular evolution is generally accelerated^20^. This usually leads to increased rate of diversification^21^, and hence the genetic structure of the populations of those species is likely to show strong geographic patterns^22^. Therefore, such species can provide a unique insight into the biogeographic history of territories like the ESSZ. Furthermore, spatial patterns in genetic variation can help us to identify those parts of the area that have conservation significance^23^. The flightless beetle, *Lethrus apterus* (Laxmann, 1770) (Coleoptera: Geotrupidae), is a good candidate as model species for such study as it inhabits steppe habitats (incl. secondary xerothermic grasslands and low impact agricultural lands) on the western part of the ESSZ. Apart from a few satellite occurrences to the east of River Volga, the distribution range spans from the Pannonian Basin on the west to the River Don and partly River Peskovatka on the east^24^ (Fig. 1). Such a distribution is typical for a Ponto-Pannonian species, and—based on the higher species diversity on the eastern, Pontic part—is commonly believed to be the result of westward colonisation event^25^. Interestingly, however, the area coincides with the steppe and forest-steppe belt in the western part, while it abruptly ends at the course of the rivers mentioned above. This border exists despite the extensive presence of similar habitats further to the east. This raises the possibility that the western part of this distribution might have a more important role in shaping the current area than previously thought. The historical formation of this peculiar Pontic-Pannonian distribution range of *Lethrus apterus* is also interesting from a speciation point of view as it represents the most wide-spread species of a lineage that most probably originated on the Eastern Balkans^26^. Additionally, *Lethrus apterus* is also an emerging model species of parental care in behavioural ecology^27–29^.

In this study, we uncover the phylogeographic structure of *Lethrus apterus* by utilising population-level sampling of a highly variable mitochondrial DNA-region and nuclear microsatellites. We address the question of how the current distribution range (Fig. 1) of this species has developed since the separation from its Eastern Balkan sister-species, *Lethrus schaumii* Reitter, 1890^26^ The understanding of the phylogeography of *L. apterus* may help us to deepen our understanding of the biogeographic history of this part of the ESSZ.

## Results

### Partitioning mitochondrial DNA diversity

**Figure 2:**
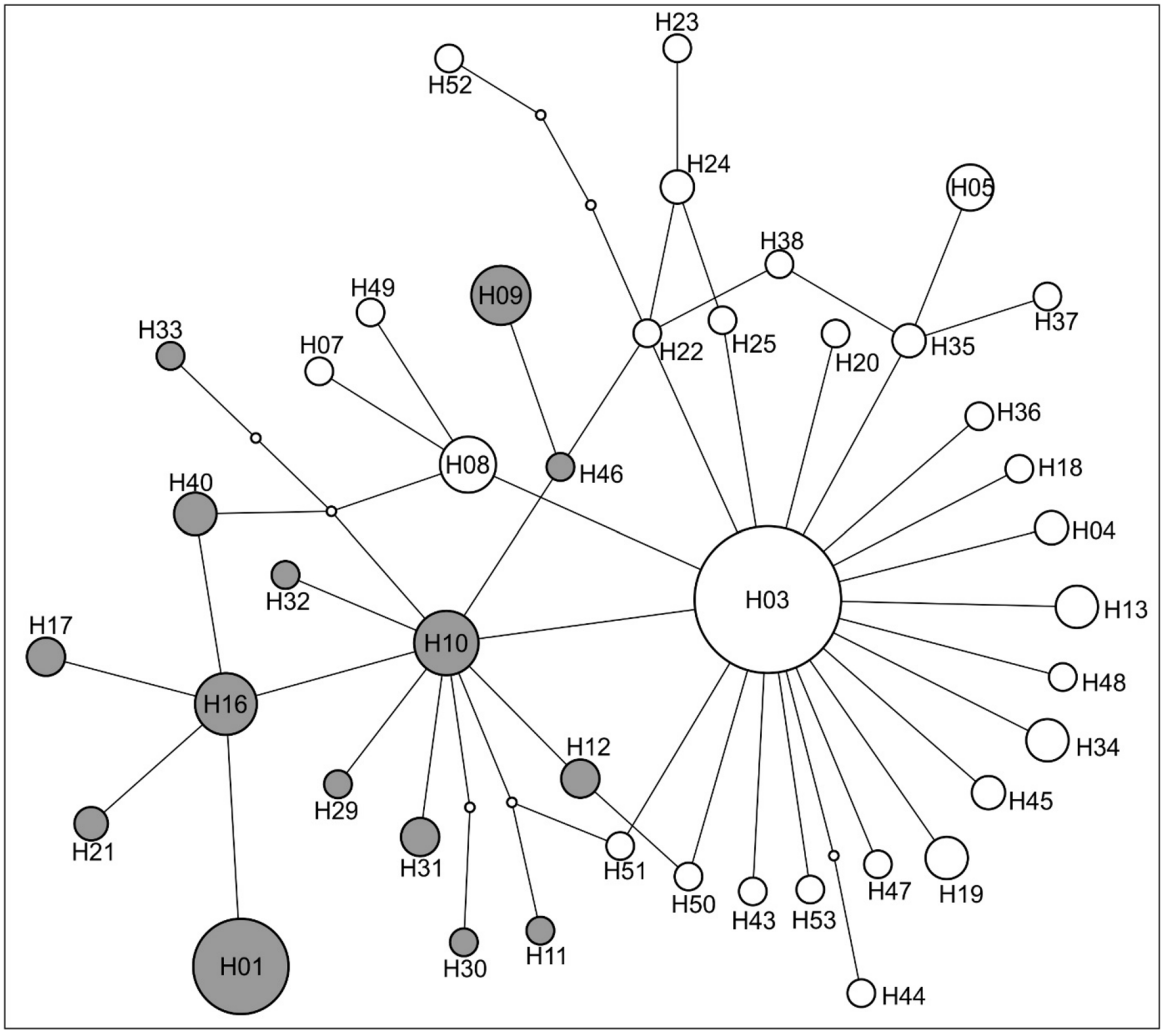
Parsimony-based haplotype-network of mitochondrial COI sequences from 36 studied *Lethrus apterus* populations obtained by TCS. Each recovered haplotype is numbered at its position on the network (either in the circle or next to it). Dots represent unsampled (extinct) haplotypes. Shaded haplotypes appear as a statistically supported (Bpp = 0.95) monophyletic clade on Fig. 3 and are geographically confined to the Hungarian Middle Range (on the western part of the distribution area).

We obtained mitochondrial cytochrome oxidase I (COI) sequences of 192 individuals from 36 populations (Table 1, Fig. 1) that were collapsed into 43 haplotypes by the parsimony-based haplotype-building algorithm (Fig. 2). The haplotype building software TCS was able to connect the network at 95% connection limit by a maximum of 12 steps. The network included a star-like structure with “haplotype 03” (H03) appearing in the centre of the network with 19 satellite haplotypes separated by only one mutation step from it.

**Table 1:** Population number, locality information of the studied populations and number of individuals included in the mitochondrial (mtDNA) and microsatellite (SSR) datasets. Country codes follow ISO standard.

Population Nr	Locality	Coordinate		Sample sizes	
		N(°)	E (°)	mtDNA	SSR
1	HU: Vizslás	48.038	19.787	5	16
2	HU: Dorogháza	47.990	19.890	0	16
3	HU: Mátraderecske	47.968	20.066	5	16
4	HU: Sirok	47.947	20.184	5	16
5	HU: Egercsehi-Szarvaskő	48.022	20.297	5	15
6	HU: Szentdomonkos	48.078	20.208	4	16
7	HU: Borsodnádasd	48.117	20.228	4	16
8	HU: Uraj	48.257	20.254	5	16
9	HU: Gömörszőlős	48.371	20.430	6	16
10	HU: Ragály	48.414	20.509	6	16
11	HU: Csobánka	47.635	18.942	6	16
12	HU: Balatonkenese	47.036	18.095	5	10
13	HU: Bakonykúti	47.243	18.201	5	16
14	HU: Tarcal	48.130	21.365	5	16
15	HU: Henye	48.150	21.352	4	16
16	HU: Macs	47.577	21.455	5	16
17	HU: Dombegyház	46.304	21.145	7	30
18	RO: Bărăteaz (Baraczháza)	45.960	21.117	0	10
19	RS: Vrdnik	45.132	19.797	5	7
20	BG: Knezha	43.552	24.164	8	15
21	BG: Ovcha mogila	43.457	25.315	10	16
22	BG: Gorna Studena	43.413	25.403	8	19
23	RO: Valea lui David	47.214	27.462	5	20
24	UA: Oleksandrivka	46.752	30.359	5	20
25	UA: Pii	49.883	31.278	5	20
26	UA: Kostychi	47.271	32.216	5	20
27	UA: Kamjanska Balka	47.048	33.580	5	20
28	UA: Bielozerka	47.382	34.423	5	20
29	UA: Starolozuvatka	48.378	35.377	5	20
30	RU: Lgov	51.624	35.321	5	5
31	RU: Liski	50.950	39.482	4	5
32	RU: Rossoshki	51.259	38.861	5	5
33	RU: Don Motorway	50.608	40.055	5	5
34	RU: Staraya Kriusha	50.190	41.108	5	5
35	RU: Gavrilsk	50.415	40.407	5	5
36	RU: Kalach-na-Donu	48.689	43.451	5	5
37	RU: Surovikino	48.538	42.788	5	5
38	RU: Trekhostrovskaya	49.140	43.864	5	5

**Figure 3:**
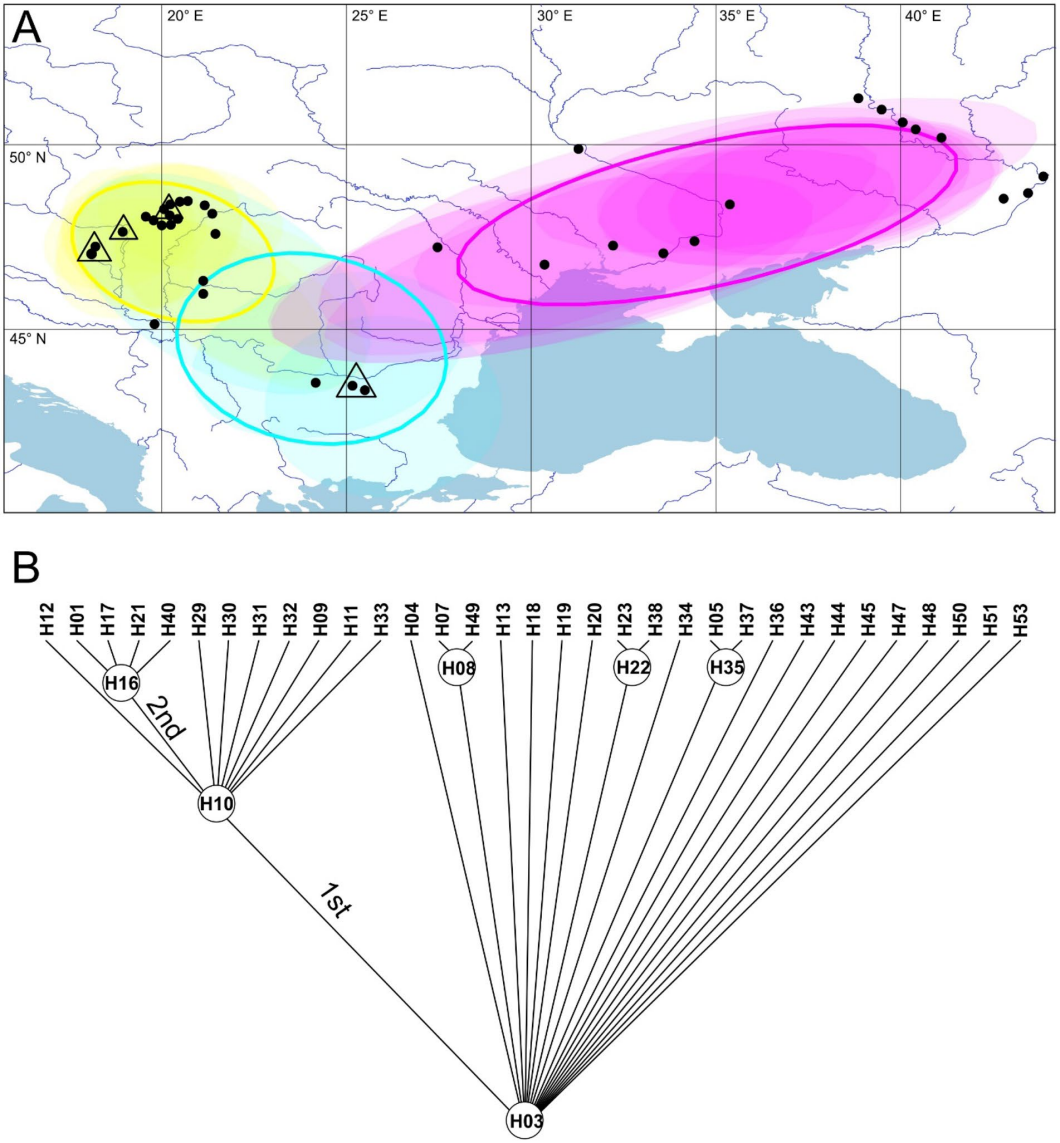
Clustering of populations based on their mitochondrial COI sequences from a Bayesian phylogeographic clustering analysis as implemented in BPEC. **(A)** The three clusters identified by the software are shown in different colours, their spatial coverage is drawn with a transparent ellipses representing the 50% highest posterior density in each run, whereas the solid lines represent posterior means. Triangles represent most likely ancestral areas with size proportional to the probability of being ancestral. **(B)** Phylogenetic relationship of mitochondrial haplotypes as reconstructed by BPEC using relaxed parsimony on a maximum a posteriori haplotype tree. Internal haplotypes are encircled, and two migration events (i.e., the emergence of a new cluster formed through the migration/dispersal/colonisation of a single haplotype leading to a new geographically distinct cluster) are also indicated next to the corresponding branch.

We studied the genetic structure of the distribution area in a Bayesian phylogeographic clustering analysis as implemented in BPEC (Fig. 3). The analysis collapsed the input sequences into 39 haplotypes on a maximum a posteriori haplotype tree. It identified the first two most probable ancestral ranges with roughly equal probability (Fig. 3A), but the ancestral node was assessed to be H03 with two migration events on the haplotype tree (Fig. 3B). Three groups of samples were identified: one group covered the eastern part of the distribution area (i.e., the Pontic Steppes) that was found repeatedly at the same geographic location (as shown by the overlay of 50% highest posterior density ellipses on Fig. 3A). The other part of the area had two clusters that are somehow overlapping although the mean posterior densities are largely separated to be confined to the Pannonian Steppes and the Bulgarian Plain. The most credible ancestral populations are either in Northern Bulgaria (populations 21 and 22—“Ovcha Mogila” and “Gorna Studena”, respectively; locality names used are given in Table 1.) or in the western part of the Hungarian Middle Range (populations 12 and 13—“Balatonkenese” and “Bakonykúti”, respectively) (Fig. 3A). Additionally, samples from the more easterly part of the Hungarian Middle Range are also identified as—albeit less probable—ancestral populations (population 7 and 11—“Borsodnádasd” and “Csobánka”, respectively).

Contrary to the common expectation, no ancestral population was identified on the Pontic Steppes: the eastern part of the area is covered by a single cluster that is found in independent runs at the same geographic area (as indicated by the overlap in 50% highest posterior density ellipses), whereas the western part of the area hosts two clusters that are less clearly defined spatially. Additionally, potential ancestral areas are indicated only at the western part of the distribution range: in Northern Bulgaria (that still belongs to the Pontic Steppes in biogeographic classifications) and in the Hungarian Middle Range.

**Figure 4:**
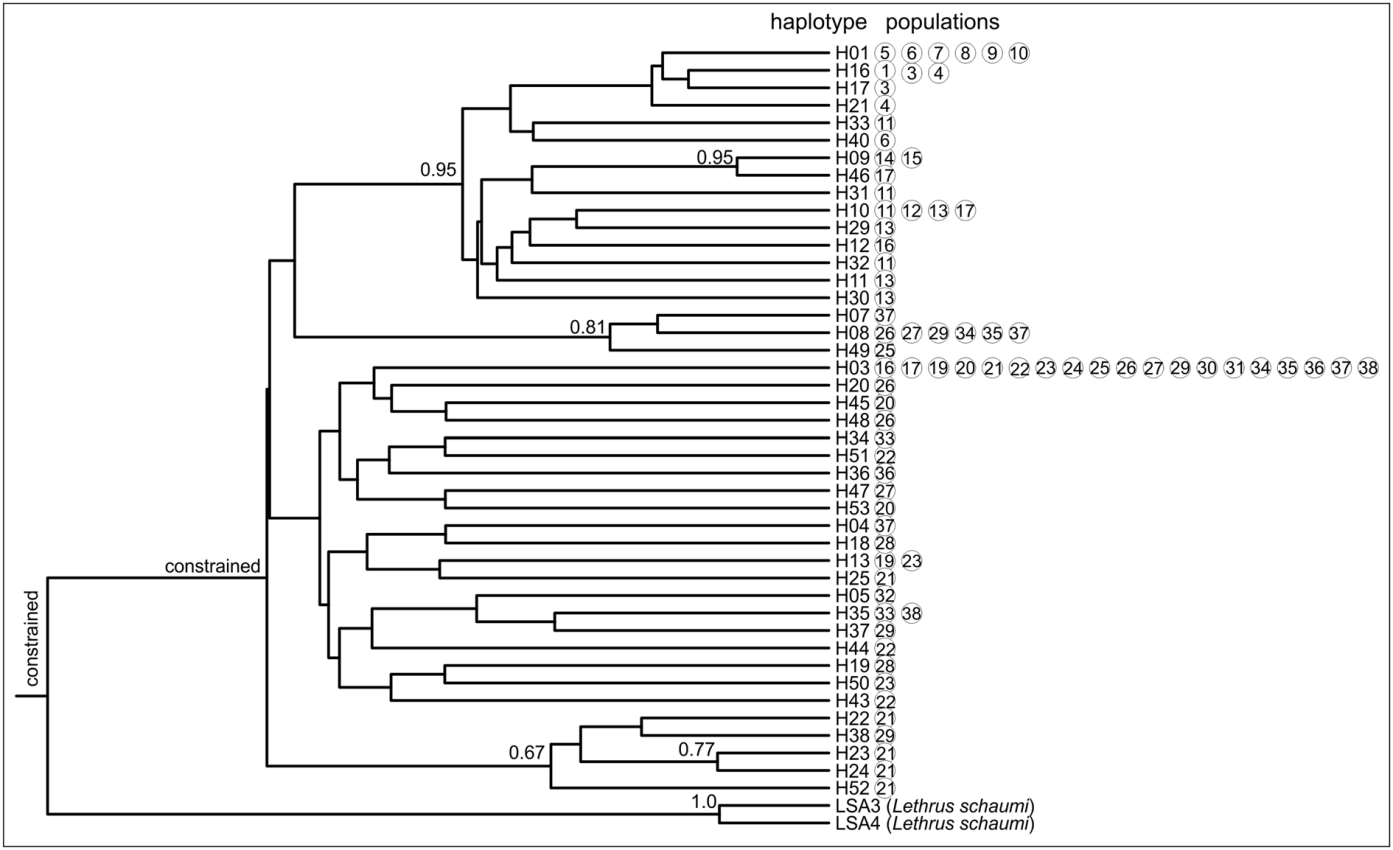
Maximum credibility phylogenetic tree resulting from the Bayesian approximation of evolutionary relationships of mitochondrial COI haplotypes of the studied *Lethrus apterus* populations (identified by their number as given in Table 1.) as estimated by the software BEAST. Haplotypes of *L. schaumii* are included as outgroup. The sister relationship of *L. schaumii* and *L. apterus* was constrained, as was the monophyly of samples of *L. apterus*. Bayesian posterior probability (Bpp) values of clade credibility is printed next to each branch receiving Bpp > 0.65 value.

We further assessed the evolutionary history of these 43 haplotypes by using a Bayesian approximation of the phylogenetic tree of haplotypes in a BEAST analysis (Fig. 4). Two haplotypes of *Lethrus schaumii* were used as outgroup from our forerunning phylogeny (GenBank accession numbers: MK558533–MK558540)^26^. The maximum clade credibility tree divided the ingroup haplotypes into four monophyletic lineages. Only one of these received strong Bayesian posterior probability (Bpp) statistical support (Bpp = 0.95) and it grouped haplotypes found exclusively in the Hungarian Middle Range within the Pannonian Basin. A second lineage received moderate support (Bpp = 0.81) and grouped haplotypes from the easternmost part of the area. A third lineage, with low statistical support (Bpp = 0.67), grouped together haplotypes from a Bulgarian locality (population 21—“Ovcha Mogila”) and one haplotype from a Ukrainian population (population 29—“Staroluzovatka”). Finally, the fourth main lineage, that did not receive statistical support, contained haplotypes that originate from populations of the whole area except the Hungarian Middle Range. The phylogenetic relationship between these four main lineages remained unresolved as none of the branches received statistical support. Apparently, these four lineages have diversified approximately simultaneously.

### Phylogeographic structure based on microsatellites

**Figure 5:**
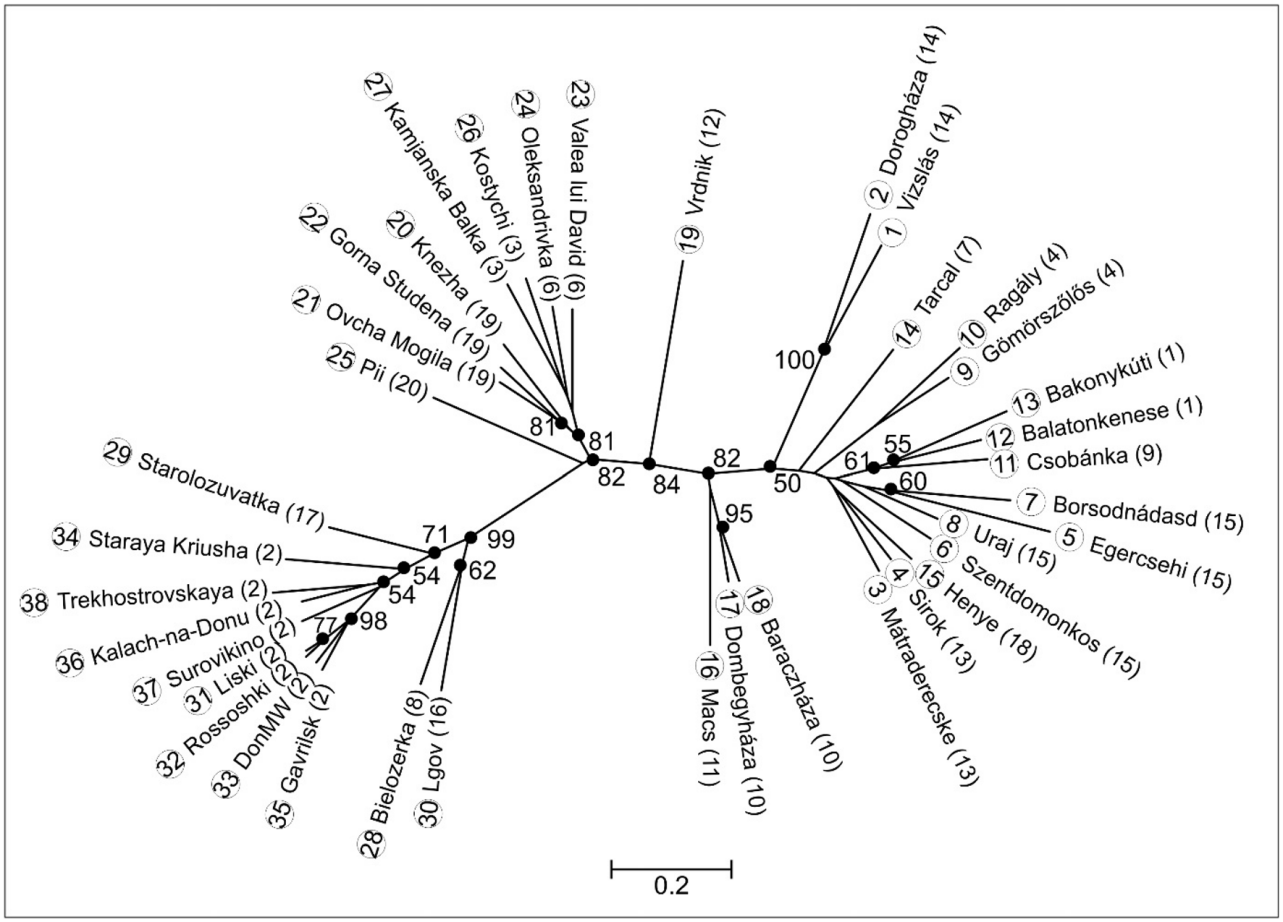
NJ-tree based on Chord distance of allelic frequencies with 100 bootstrap support displayed at supported nodes. Unsupported nodes (bs < 50%) are not dotted. Each population is identified by the name and its population numbers as shown in Table 1, which is followed by genetic clustering number in parentheses as identified by Geneland genetic clustering analysis. Scale bar stands for 0.2 unit of Chord distance.

From 38 populations we genotyped 531 individuals for 16 microsatellite (SSR) loci^30^. None of the loci showed departure from Hardy–Weinberg equilibrium (HWe) and we did not observe the presence of null-alleles. Therefore, all loci were used in downstream analyses without correction. The Neighbour-Joining (NJ) tree based on Chord-distance of allelic frequencies showed genetic groups that mirror geography (Fig. 5): the left part of the tree contained all Pontic Steppes populations east of River Dnieper separated along a highly supported (bootstrap, bs = 99%) branch. Further, supported splits separated a group of populations from the Bulgarian Plain and the Pontic Steppes west of River Dnieper (bs = 82%), but all Pannonian Steppe populations were also clearly separated (bs = 82%). As a clear intermediate population, the Serbian sample (population 19—“Vrdnik”) was placed between these two latter lineages (bs = 84%). The populations of the Great Hungarian Plain (populations 17, 18 and 16—“Dombegyháza”, “Baraczháza”, “Macs”, respectively) showed separation with marginal statistical support (bs = 50%), and populations of the western part of the Hungarian Middle Range, to the west of River Danube, (populations 13, 12 and 11—“Balatonkenese”, “Bakonykuti” and “Csobánka”, respectively) also showed separation (bs = 61%). A statistically significant (bs ≥ 81%) grouping was formed by samples from the Bulgarian Plain (populations 20, 21 and 22—“Knezha”, “Ovcha Mogila” and “Gorna Studena”, respectively), and populations from the eastern periphery of the distribution range in Russia close to River Don (populations 31, 32, 33 and 35—“Liski”, “Rossoshki”, “Don Motorway” and “Gavrilsk”, respectively) that are significantly separated (bs = 98%). Additional groupings were formed by geographically close populations (e.g., populations 1 and 2—“Vizslás” and “Dorogháza”; populations 9 and 10—“Gömörszőlős” and “Ragály”; populations 17 and 18—“Dombegyháza” and “Baraczháza”, respectively).

Large rivers were found to be important indicators of significant separations. The populations from the eastern shore of River Dnieper are separated significantly (bs = 99%) on the NJ-tree (Fig. 5) from those on the west bank; the populations on the Bulgarian Plain are separated from the populations of the Pontic Steppes north of River Danube by bs = 81%; and the Serbian population on the southern bank of River Danube is separated from other Pannonian Steppe populations with a similarly high support (bs = 84%); populations on the Great Hungarian Plain are separated from the populations of the Hungarian Middle Range north of River Tisza with high support (bs = 82%). Interestingly, the separation of the populations from the western part of the Hungarian Middle Range, which is split into two sides by River Danube, is less pronounced (bs = 61%). Similarly, River Don causes apparently no separation at the very eastern margin of the area.

The Geneland clustering analysis identified in all five runs the same, 21 most probable genetic clusters that correspond to the grouping based on allelic frequencies (Fig. 5). Interestingly, Geneland found eight genetic clusters (Fig. 5: clusters 15, 9, 1, 13, 18, 4, 7, 14) in the Hungarian Middle Range (across ca 5000 km^2^ area), whereas only four were found on the vast area (ca 90,000 km^2^) of the Pontic Steppes east of River Dnieper (Fig. 5: clusters 2, 8, 16, 17).

**Figure 6:**
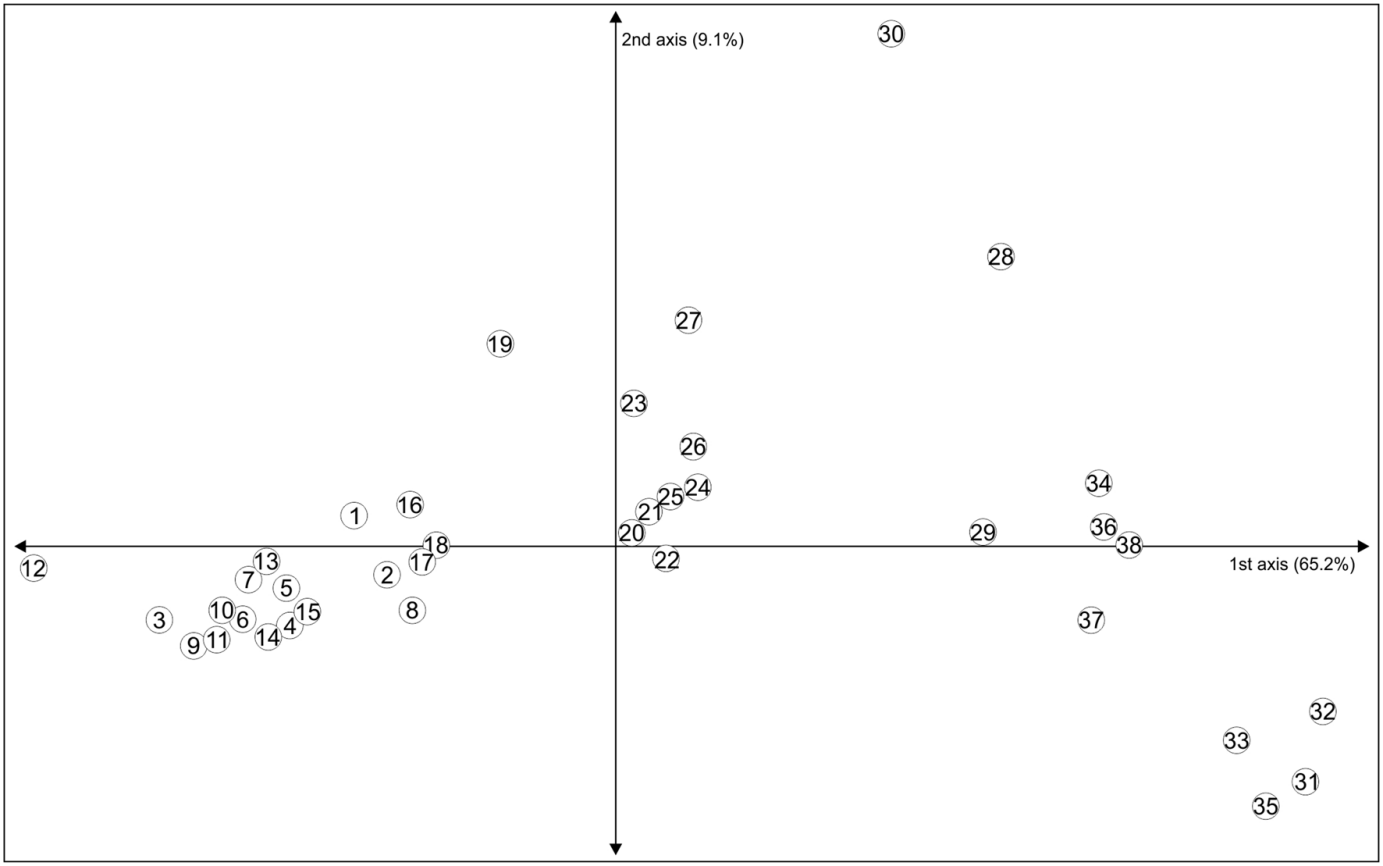
Genetic differentiation (Gst) of the studied populations visualised as a Principal Coordinate Plot showing the first two axes that cumulatively cover 74.3% of total variance. Populations are identified by their number as given in Table 1.

**Figure 7:**
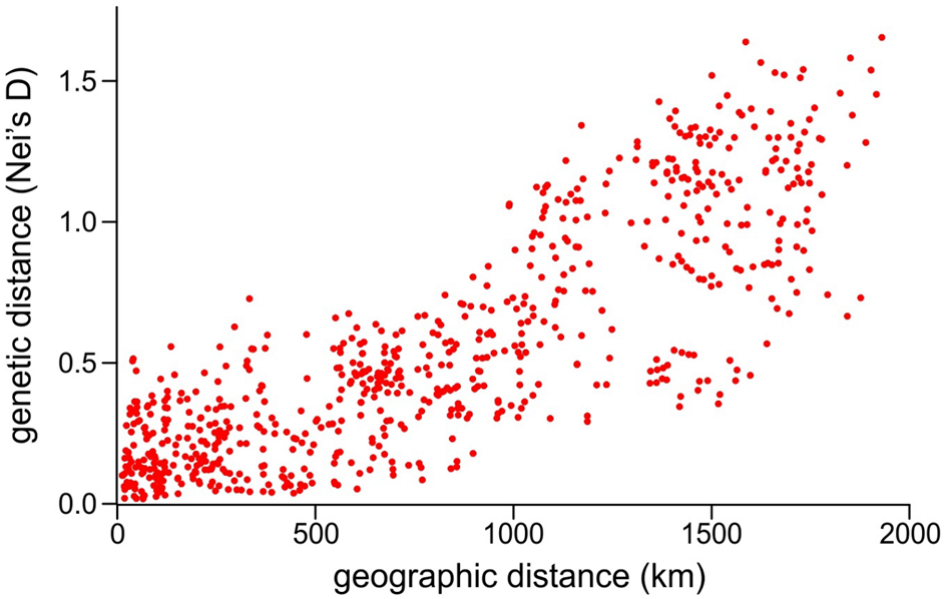
Test of isolation-by-distance at the population level (Mantel-test, p < 0.001).

The genetic differentiation of the studied populations was visualised in a Principal Coordinate Analysis (Fig. 6). The first axis—that separated the populations along an east-to-west geographic gradient—accounted for a remarkable 65.2% of total variability. The genetically most differentiated population group was formed by the Russian populations at the eastern edge of the distribution range close to River Don (populations 31–38 on Fig. 1). Also differentiated are other populations east of River Dnieper (populations 28–30 on Fig. 1). Some differentiated populations showed a remarkable divergence from all the rest, e.g. population “Balatonkenese” (population 12 on Fig. 1) or “Lgov” (population 30 on Fig. 1). Otherwise, the genetic differentiation reflects geography (cf. Fig. 1). In line with this pattern, we found a very strong isolation-by-distance (IBD) pattern in our dataset: the genetic distance matrix showed a very significant correlation with the pairwise geographic distances (Mantel-test, p < 0.001; Fig. 7).

**Figure 8:**
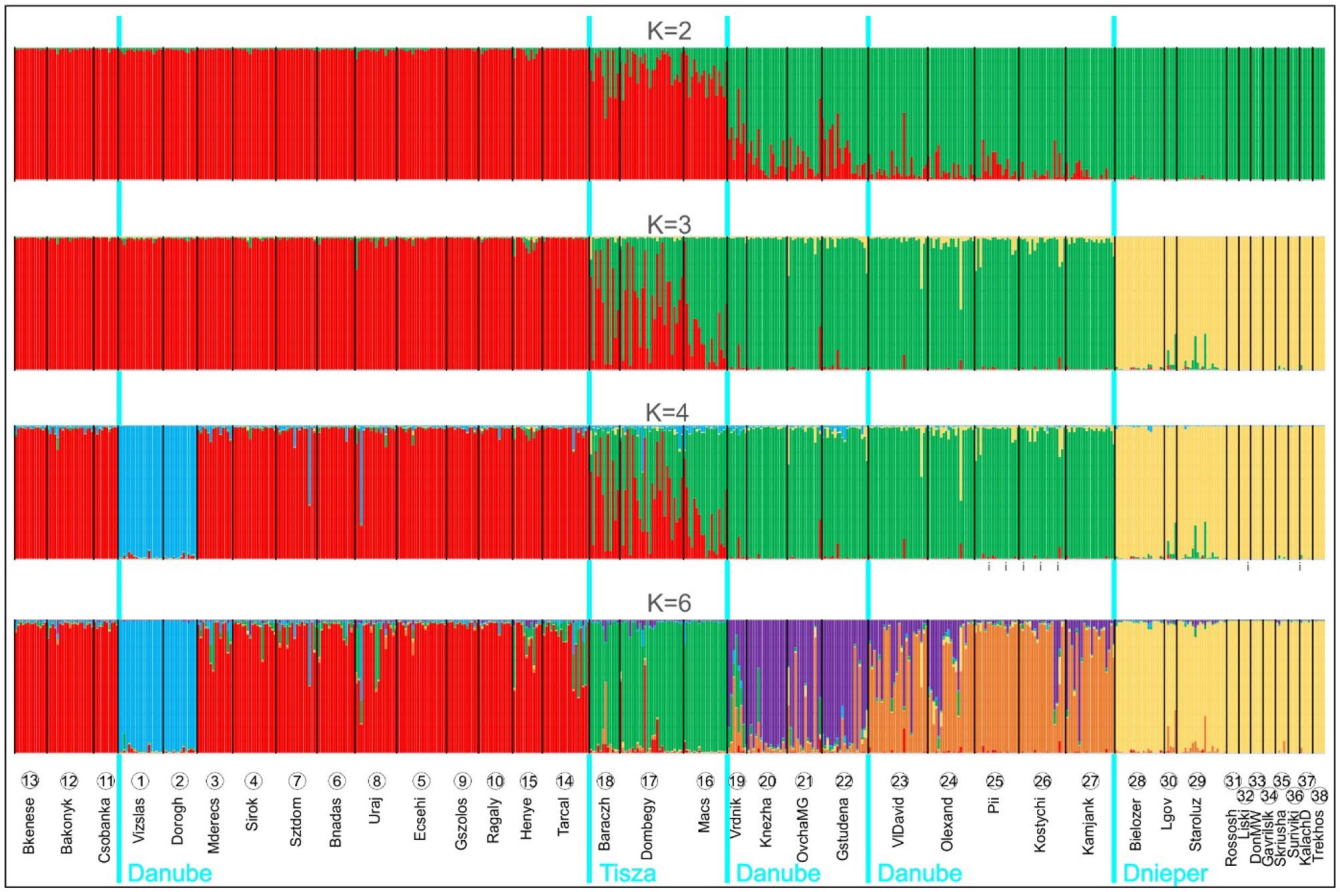
Hierarchical Bayesian clustering of individuals into K = 2, 3 and 4 and 6 groups by the software STRUCTURE. Main rivers separating the population groups are indicated on the map by a light blue line and the name of the river. Populations, arranged along a longitudinal gradient, are indicated below the bar plots at the corresponding place by their number as given in Table 1 and their name.

Finally, we run STRUCTURE to study population structure on the basis of our microsatellite dataset. As delta K steeply decreased indicating, according to Evanno’s method, the relatively best K at K = 2 (see also^31^), we decided to visualise individual assignment plots at hierarchical level for K = 2, 3, 4, and 6 (Fig. 8). At K = 2 populations from the Pannonian Steppes were included in one group, and populations of the Pontic Steppes (including the populations of the Bulgarian Plain) were included in the other group. Somehow intermediate was the Serbian population (population 19—“Vrndik”). Interestingly, the populations from the Hungarian Middle Range showed almost admixture-free genotypes, whereas the populations east of River Dnieper showed similarly “pure” genotypes of the other sort. At the next hierarchical level, K = 3, the latter group separated as a third group. The populations of the Great Hungarian Plain (populations 16, 17, and 18) expressed more admixed composition (i.e., they become apparently “more intermediate”). Surprisingly, only two populations from the Hungarian Middle Range (populations 1 and 2—“Vizslás” and “Dorogháza”) split off at K = 4. Although further clustering of samples are not validated by Evanno’s method (but see^31^), we found K = 6 to be informative (Fig. 8). If supposing the existence of K = 6 genetic clusters, the Hungarian Middle Range remains relatively homogenous (while the two above populations still separate), a rather homogenous group of samples from the Great Hungarian Plain emerges, which is followed by the somehow homogenous group of populations from the Bulgarian Plain and Serbia (all south of River Danube). This is followed by a group of populations west of River Dnieper with the westernmost samples showing signs of admixture to the previous group. Finally, we find the homogenous group of populations east of River Dnieper including those few populations (33, 34, 35) that managed to cross the next large river to the east, River Don. In accordance with the results of the analysis based on Chord genetic distance (Fig. 5), the boundaries of population-groups inferred from the STRUCTURE analysis clearly coincide with large rivers, especially at higher hierarchical levels (Fig. 8).

## Discussion

Recent analyses^19^ have shown that western peripheral populations of some widely distributed steppic species can represent old relicts with high conservation value. In this respect, a comprehensive meta-analysis^18^ has already suggested a similarly high value of the Pannonian Steppes in preserving genetic diversity of steppic species. In this study we examine another example of steppe organisms by investigating the phylogeographic patterns within *Lethrus apterus*, a flightless beetle species inhabiting steppes and dry grasslands. Both the mitochondrial COI and the nuclear SSR indicate a very uneven distribution of genetic variation of *Lethrus apterus* throughout its whole distribution range: the regional genetic variability at the western part of the area (i.e., the Pannonian Steppes and the Bulgarian Plain) is remarkably higher than on the east (i.e., the Pontic Steppes). Also, the explicit ancestral area assessment of the BPEC analysis found these two western areas to be possible ancestral area of the species (Fig. 3).These results strongly argue for the ancestral area of *Lethrus apterus* might have laid at the western part of the current area, whereas it seems safe to conclude that this beetle is a recent arrival on the Pontic Steppes.

This finding is further supported by finer structures found in our analyses. The presence of a highly supported mitochondrial lineage (Fig. 4) in the Hungarian Middle Range can also be the consequence of the relatively long-term presence of these populations on an evolutionary time-scale not excluding some possible bottlenecks these populations went through during restrictive fluctuations of their range and fragmentation of habitats within this region. The haplotypes, geographically confined to this area, are placed on a rather well-structured part of the haplotype network (Fig. 2), whereas the rest of the network is dominated by the central haplotype “H03” (found in 64 samples from the Pontic Steppes and the Great Hungarian Plain) that form a star-like structure with relatively long external branches. Such phylogeographical structure is usually indicative of relatively recent expansion^32^ where the central, geographically most widespread haplotype has several, low-frequency satellite haplotypes (usually one mutation step away from the central one). Taking the frequently elevated evolutionary rate of flightless beetles^20^ into consideration, this finding can be a result of relatively recent, independent isolation from the common haplotype probably as a result of post-dispersal diversification.

Nuclear microsatellites (SSRs) show further evidence of the evolutionary importance of the western part of the area. Genetic distance (Fig. 6) reflects geography with the main, statistically supported groups arranged according to a longitudinal gradient. Whereas this can simply be the reflection of the strong isolation-by-distance found in our analysis (Fig. 7), the remarkably higher number of genetic clusters (Fig. 5) in the Hungarian Middle Range can be indicative of long-existing populations that had enough time to diversify into several groups. On the contrary, the remarkably homogenous eastern part of the area is again an indication of a relatively recent arrival of the species. Hierarchical Bayesian clustering of SSR variability (Fig. 8) further strengthen our view on the uneven distribution of genetic variability throughout the distribution area of *Lethrus apterus*.

This evolutionarily recent arrival of populations to the easternmost part of the area is also clearly indicated by the genetic differentiation of populations (Fig. 6.): populations from the eastern part of the area (especially those on the very eastern edge) show the highest level of genetic differentiation from the rest of the populations. Such strong differentiation of a group of populations can follow from a recent colonisation that suffered from founder effect—a special case of genetic drift caused by a bottleneck in population size because of the founding of a new population by a few individuals^33^. Although several outlier populations can also be spotted on the figure, we interpret those as a result of recent bottlenecks these populations (e.g., “Balatonkenese”, “Lgov”, etc.) went through. Such founder effects could happen when the pioneer population manages to surmount an important barrier; such as a large river which must be a difficult-to-surpass barrier for a flightless beetle. And indeed, populations groups on either side of River Dnieper or River Danube are usually separated by highly supported branches indicative of significant genetic distances on our NJ-tree of genetic distance (Fig. 6). The role of floodplains as barriers can be especially important for this subterranean species as it nests and overwinters underground, and can consequently be especially sensitive to early spring flooding. All these separations, and the coincidence of the remarkable genetic differentiation of several of these population groups on the Principal Coordinate Analysis plot (Fig. 6) clearly highlight the important role of large rivers as barriers for this flightless beetle species. The same was found by Kajtoch et al.^22^ who found that River Vistula is an important barrier for a flightless weevil species. Similarly, large rivers are also important barriers for a subterranean rodent genus (*Nannospalax*) in the Pannonian Basin^34^.

However, we also found instances where the isolating nature of large rivers are apparently weaker. Such an exemption is River Danube in Hungary, where the separation of the populations on the western part of the Hungarian Middle Range is less pronounced. As this river has a much narrower floodplain where it crosses the Hungarian Middle Range^35^, supposedly the crossing of Danube was more feasible than the much wider floodplain of River Tisza. Another remarkable exemption is River Don on the very eastern part of the area (Fig. 1). Nevertheless, the recent arrival at this large river and the recent successful crossing of it by the species can offer a very likely explanation to the observed pattern. This is further corroborated by the extensive presence of apparently suitable habitats on the left bank of River Don, but the species was able to extend its continuous area east only up to the next river: River Peskovatka.

Based on the above arguments, we conclude that the ancestral area of *Lethrus apterus* might have laid at the western part of the current area. The Hungarian Middle Range, which is a relatively low elevation mountainous-hilly area surrounded by an extensive foothill region characterised by elevated values of relative relief energy^35^ and is part of the forest-steppe zone^4^, could have repeatedly served as a refugium during the Upper Pleistocene including the last glacial maximum. This is further supported by the presence of significant steppe communities and plant species in the Pannonian Plain in the geological past^36–38^.

Notwithstanding, BPEC analysis also indicated the possible presence of an ancestral population on the Bulgarian Plain. Whereas we do not have further evidence to support the presence of an ancestral area here, we cannot rule out the primary role of this territory in the colonisation of *Lethrus apterus*. As the sister species, *L. schaumii*^26^, lives in North-Eastern Greece, formal logic would imply that the Bulgarian Plain was colonised first. From here, the species might have colonised towards the north-west and reached the Pannonian Region. In a subsequent unfavourable period, the species could have retreated from most of its area, but survived in the Hungarian Middle Range (as indicated by the findings presented above), but it could also have survived in the Bulgarian Plain. These two areas then became the core areas of colonisation of the current distribution area. The Hungarian population could colonise the Great Hungarian Plain where it could have mixed with the lineage surviving in the south. Clearly, the Bulgarian group of populations managed to colonise the Pontic Steppes. This marching eastwards must have been hindered by the large, north–south flowing rivers of Eastern Europe, especially Rivers Dnieper and Don, which resulted in reduced number of colonisation events to the east banks.

An alternative scenario can also be drawn from these results. It is possible that the only surviving populations of the unfavourable period were those in the Hungarian Middle Range, and the species has simply marched eastwards from this ancestral area via the Lower Danube region (located on the border between Serbia and Romania). This scenario is supported by the strong isolation-by-distance we found, and the presence of the single, well-differentiated mitochondrial lineage that is confined to and trapped in the Hungarian Middle Range. Whatever was the actual history of the populations, both of the above alternatives of colonisation history share a common regularity: the Pontic Steppes was recently colonised from the west. Therefore, we can safely conclude the western origin of this Pontic-Pannonian steppe species with a rear edge at the western part, and a leading edge on the east. This is a somewhat surprising discovery given the higher density of current *Lethrus* populations on the east.

The importance of the Pannonian Basin as a refugium for certain steppe plants have already been suggested^18,39,40^. Although numerous examples of phylogeographic analysis of Ponto-Pannonian steppe species are known in the literature^41–45^, most of these focus their sampling on a smaller part of the whole distribution area, which may undermine phylogeographic reconstruction of the analysed species^42,46^. Therefore, we must be cautious in comparing our phylogeographic results with similar results on other steppe species, but the general acceptance of the presence of a steppe refugium in the western part of the area for certain steppe species forecast similar colonisation patterns for—at least some—steppe species: from the west to the east, from the current periphery to the eastern zonal range (“centre”). Somewhat similar eastward colonisation from a Balkan (not a Pannonian) refugium has been demonstrated for two animals inhabiting dry grasslands: the butterfly *Melitaea ornata*^47^ and the land snail *Caucasotachea vindobonensis*^48^ reached the Pontic Steppes from the south-west.

If we suppose a more general west-to-east colonisation pattern of steppe species from the current periphery to the extant main area (i.e., from the extrazonal part of the range to the zonal one), this can also raise the question of the relative role of an Eastern Balkan and/or a Pannonian refugium in this process. To further decipher the detailed evolutionary history of populations on the western edge of the distribution area, both in general and in this specific species, a deeper phylogenetic resolution is needed. Reduced genomic representation sequencing (e.g., RADseq) can easily outperform both marker systems^49^ used in this study and could validate one of the two equally parsimonious hypotheses about western refugia for this species.

There is also a clear conservation message here. As postulated by the “rear edge—leading edge” hypothesis^23^, the western populations deserve high conservation priority as rear edge populations were shown to possess more genetic resources and thus, they can source future population expansion. In contrast to the eastern part of its distribution area, where this species is rather common^50^, *Lethrus apterus* has recently experienced a steep decline in Hungary that qualified this species to be protected in the country^51^. In light of our above results this protection can be fully justified and not just as a declining species of the steppes but also as important core (“rear edge”) populations of the species. The remnant steppe patches and their characteristic steppe species can be important flagships of Pannonian conservation as these are not just interesting steppe relicts, but can also be important sources of the current genetic diversity. In this respect, the Pannonian Steppes (incl. the colline level of the Hungarian Middle Range) can play a similar role to what was found by Kirschner et al.^19^ for the steppe relicts at the range periphery of the European Alps.

In summary, our study yielded an insight into the evolutionary history of this Pontic-Pannonian steppe species, and thus can help to better understand the evolutionary history of this part of the Euro-Siberian steppe zone. Given the generality of such distribution type of steppe organisms^17^, this example may serve as a general model for future, range-wide phylogeographies on steppe species with a similar distribution. These results also suggest the prominent role of areas on the western border of the steppe zone in the (re)colonisation of its more easterly parts.

## Methods

### Sample collection and DNA extraction

Our field samples represent the entire distribution area of *Lethrus apterus* (Fig. 1; Table 1). We aimed to represent the within population genetic diversity, thus, we included 4–10 individuals (mean 5.05, n = 36 populations) for the analysis of mitochondrial DNA and 5–20 specimens (mean 13.97, n = 38 populations) for the analysis of nuclear microsatellites (SSR). Specimens were euthanised on the field and stored in 96% ethanol at -20 °C prior to DNA isolation. As this species is protected in Hungary, sample collection was approved by the North Hungarian Inspectorate for Environment Protection and Nature Conservation (No. 9007-8/2014). Total genomic DNA was extracted from the tarsus of the beetles as detailed in Rácz et al.^30^, then DNA isolates were stored at −20 °C.

### Analyses of mtDNA sequence variation

Amplification of the mitochondrial cytochrome oxidase I (COI) gene followed the protocol of Tóth et al.^26^, then amplicons were sequenced using a commercial service provider (Macrogen Inc., South Korea). Raw electropherograms were checked by eye for obvious sequencing errors which were corrected manually. For some analyses the outgroup sequences of *Lethrus schaumi* were obtained from the dataset of Tóth et al.^26^ (GenBank accession numbers: MK558533–MK558540). Sequences were aligned using MUSCLE^52^, then the alignment was checked for erroneous internal stop codons.

Individual sequences were collapsed into haplotypes and the most parsimonious relationship between them was inspected by calculating the 95% connection limit as implemented in TCS v.1.21^53^.

The variability discovered in the mitochondrial dataset was partitioned into phylogeographic clusters by a Bayesian clustering method used by the R^54^ package BPEC^55^. This method assumes that population substructure is a result of migration and defines clusters as geographically aggregated haplotype groups. BPEC was run for five million generations saving 10,000 iterations in total with a parsimony relaxation value of two and setting the maximum number of migrations to four.

The phylogenetic relationship of haplotypes was reconstructed using BEAST v.2.6.0^56^. For this analysis we picked one random representative of each haplotype and included *Lethrus schaumi* as outgroup species. BEAST was run twice for 100 million generations saving every 10 000th iteration. Usage of the optimal substitution model was ensured by running bModelTest^57^ simultaneously. In both runs we assumed a strict molecular clock with the Yule process of speciation. The two runs were checked for convergence using Tracer v.1.6.0 and combined with the LogCombiner module of BEAST. The maximum clade credibility (MCC) tree was assessed and drawn by the TreeAnnotator module of BEAST.

### Phylogeographic analyses using SSRs

To genotype SSR alleles of 16 nuclear loci we applied the protocol detailed in Rácz et al.^30^. Allele length were scored by the same person (R. Rácz) using Peak Scanner v.1.0 (Applied Biosystems).

The presence of null alleles was checked by using Micro-Checker v.2.2.3^58^. Population genetic statistics including allele frequencies of populations (AFP), observed and expected heterozigosity (Ho and He), deviation from the Hardy–Weinberg equilibrium (HWe) and genetic differentiation (Gst) were calculated by using GenAlex v.6.5b3^59^. The Neighbour-Joining (NJ) phylogram based on pairwise Chord-distance of raw allelic frequencies between populations was constructed and visualised with PAST v.2.17^60^ using 100 bootstrap replications to test the support of branches. Isolation by distance (IBD) was tested with the mantel.randtest function of the R package ade4 v.1.7^61^ using 10 000 permutations on the pairwise unbiased Nei genetic distance calculated by GenAlEx and pairwise geographic distances calculated from the geographic coordinates by the R package fossil v.0.4.0^62^.

Bayesian clustering based on SSR multilocus genotypes was achieved using two different approaches. First, we ran STRUCTURE v.2.3.5^63^ with K values ranging from one to 30 with a chain length of five 500,000 steps and a burn-in of 100,000 steps. To assess convergence between runs each K value was run five times using ParallelStructure v.1.0^64^. Optimal clustering was checked by Evanno’s method as implemented in Structure Harvester (http://taylor0.biology.ucla.edu/structureHarvester/). As suggested by Janes et al.^31^, we checked multiple K values to assess the hierarchical clustering of populations. Our second approach used the spatial clustering algorithm of the R package GENELAND v.4.0.6^65^, which is able to assign individuals to genetic clusters based on their multilocus genotype and spatial placement. For this analysis we used the correlated frequency model and set the length of the Markov Chain to two million with a thinning of 200 and maximum number of nuclei of 1593, three times the number of individuals. We assumed a minimum K value of one and a maximum of 30. Again, we checked convergence of runs by running the analysis five times, then evaluated the run that had the highest average posterior probability.

## Supplementary Information

Supplementary Information.

## Data Availability

DNA sequences generated for the study were submitted to GenBank and available under the accession numbers MW402898–MW402940. Raw microsatellite genotypes are available as a Supplementary Table.
